# The mechanics of implementation strategies and measures: advancing the study of implementation mechanisms

**DOI:** 10.1186/s43058-022-00358-3

**Published:** 2022-10-22

**Authors:** Cara C. Lewis, Predrag Klasnja, Aaron R. Lyon, Byron J. Powell, Rebecca Lengnick-Hall, Gretchen Buchanan, Rosemary D. Meza, Michelle C. Chan, Marcella H. Boynton, Bryan J. Weiner

**Affiliations:** 1 Kaiser Permanente Washington Health Research Institute, Seattle, WA USA; 2 School of Information, University of Michigan, Ann Arbor, MI USA; 3 Department of Psychiatry and Behavioral Sciences, University of Washington, Seattle, WA USA; 4 Center for Mental Health Services Research, Brown School, Washington University in St. Louis, St Louis, MO USA; 5 Center for Dissemination & Implementation, Institute for Public Health, Washington University in St. Louis, St. Louis, MO USA; 6 Division of Infectious Diseases, John T. Milliken Department of Medicine, School of Medicine, Washington University in St. Louis, St. Louis, MO USA; 7 Division of General Medicine and Clinical Epidemiology, University of North Carolina at Chapel Hill School of Medicine, Chapel Hill, NC USA; 8 NC TraCS Institute, University of North Carolina at Chapel Hill, Chapel Hill, NC USA; 9 Lineberger Comprehensive Cancer Center, University of North Carolina at Chapel Hill, Chapel Hill, NC USA; 10 Department of Global Health, University of Washington, Seattle, WA USA

**Keywords:** Mechanisms, Implementation Science, Measures, Causal pathway diagrams, Theory, Determinants

## Abstract

**Background:**

There is a fundamental gap in understanding the causal mechanisms by which strategies for implementing evidence-based practices address local barriers to effective, appropriate service delivery. Until this gap is addressed, scientific knowledge and practical guidance about which implementation strategies to use in which contexts will remain elusive. This research project aims to identify plausible strategy-mechanism linkages, develop causal models for mechanism evaluation, produce measures needed to evaluate such linkages, and make these models, methods, and measures available in a user-friendly website. The *specific aims* are as follows: (1) build a database of strategy-mechanism linkages and associated causal pathway diagrams, (2) develop psychometrically strong, pragmatic measures of mechanisms, and (3) develop and disseminate a website of implementation mechanisms knowledge for use by diverse stakeholders.

**Methods:**

For the first aim, a combination of qualitative inquiry, expert panel methods, and causal pathway diagramming will be used to identify and confirm plausible strategy-mechanism linkages and articulate moderators, preconditions, and proximal and distal outcomes associated with those linkages. For the second aim, rapid-cycle measure development and testing methods will be employed to create reliable, valid, pragmatic measures of six mechanisms of common strategies for which no high-quality measures exist. For the third aim, we will develop a user-friendly website and searchable database that incorporates user-centered design, disseminating the final product using social marketing principles.

**Discussion:**

Once strategy-mechanism linkages are identified using this multi-method approach, implementation scientists can use the searchable database to develop tailored implementation strategies and generate more robust evidence about which strategies work best in which contexts. Moreover, practitioners will be better able to select implementation strategies to address their specific implementation problems. New horizons in implementation strategy development, optimization, evaluation, and deployment are expected to be more attainable as a result of this research, which will lead to enhanced implementation of evidence-based interventions for cancer control, and ultimately improvements in patient outcomes.

**Supplementary Information:**

The online version contains supplementary material available at 10.1186/s43058-022-00358-3.

Contributions to the literature

- This protocol paper surfaces four critical challenges to advancing the study of implementation mechanisms
- This paper details a series of studies to address these four challenges
- Factors implicated in an implementation strategy causal pathways are defined and methods for articulating them are provided

## Background

Implementation science is poised to make a transformative advance by illuminating the causal mechanisms through which implementation strategies influence evidence-based practice (EBP) implementation, care delivery, and, ultimately, patient outcomes. Knowing *how* strategies work—that is, knowing their mechanisms—will facilitate identifying the barriers and facilitators that specific strategies effectively address, and the conditions under which strategies work well, poorly, or not at all. Likewise, implementation strategies, including multilevel or multicomponent ones, could be optimized by adding or strengthening components that activate mechanisms to influence key barriers while eliminating components that do not. Knowledge from mechanisms-focused implementation research will offer practitioners guidance about which implementation strategies to use in which contexts.

To address calls from funders, journals, leaders in cancer control [1, 2], and the implementation research field at large [3–5], we have identified critical challenges that must be addressed to inform when and in what contexts specific implementation strategies should be used and when they should not. First, many strategies are underspecified in their core components [6, 7] which makes it difficult to identify mechanisms of change [7] and to replicate successful approaches. Second, little evidence of implementation strategy mechanisms exist. A 2016 systematic review of implementation mechanisms in mental health found no empirically supported mechanisms in nine studies [8]. Another systematic review of implementation mechanisms examined 46 studies across health domains and found that none identified a mechanism for even a single strategy [9]. Third, there are few testable causal accounts of how implementation strategies operate, the proximal and distal outcomes they impact, and the preconditions (i.e., factors that must be in place to activate mechanisms) and moderators that influence their effectiveness. Fourth, reliable, valid, pragmatic measures of implementation mechanisms are sorely lacking [10], impeding evaluation of implementation strategies and their causal pathways. Furthermore, existing measures often lack desirable psychometric qualities.

### Current study

This manuscript presents the protocol for a three-year research project that seeks to address these critical issues. Our research project will employ a structured approach that integrates theory, empirical literature, and qualitative inquiry with expert panel methods across a series of studies that build on and validate the ones prior. We draw on Agile Science [11], a new approach for developing and studying behavioral interventions that borrows concepts from computer science and offers tools for formulating causal pathway diagrams, to articulate plausible mechanisms for commonly used strategies [12, 13], clarifying how implementation strategies operate. Three aims guide this research project.

- Aim 1: Build a database of causal pathway diagrams of implementation strategy functioning. We will first identify plausible strategy-mechanism linkages by interviewing 30 principal investigators of implementation scienced-focused National Institute of Health-funded grants in cancer control or mental health. We, the investigative team, will then develop casual pathway diagrams (CPD; i.e., graphical depictions of factors implicated in strategy operations) for 30 commonly used implementation strategies [10]. Finally, we will affirm the plausibility and strength of strategy-mechanism linkages, and completeness of the CPD, for the 30 strategies by soliciting feedback from diverse stakeholders who are experts in their use of each strategy.
- Aim 2: Develop reliable, valid, pragmatic measures of six implementation mechanisms that operate at intrapersonal, interpersonal, or organizational levels, using our established rapid-cycle measure development and testing procedures [14].
- Aim 3: Develop and disseminate an interactive website repository of implementation mechanisms knowledge using iterative user-centered design and social marketing principles.

## Methods

This project is a series of interrelated studies and activities intended to yield causal pathway diagrams of common implementation strategies, methods and associated toolkits, and measures to be disseminated through a user-friendly website. Our approach ensures the application of the most appropriate methods for rigorous and efficient study completion for each aim, with each activity serving as a validation check for preceding activities (Table 1).

**Table 1:** Research strategy overview

Aim	Inputs	Methods	Outcomes
1a	Expertise of principal investigators	Semi-structured interviews	Specified strategies, plausible mechanisms
1b	30 commonly used strategies Extant literature	Causal pathway diagramming	Causal pathway diagrams
1c	Diagrams of 30 commonly used strategies	Expert panel validation process	Confirmed strategy-mechanism linkages
2	Existing measures, theory	Rapid measure development	6 measures of mechanisms
3	Causal pathway diagrams 6 new measures	User-centered design, social marketing	User-friendly website, relational database, dissemination effort

### Aim 1a: Identify strategy-mechanism linkages in US National Institutes (NIH)-funded research

#### Design and sample

This study will involve conducting semi-structured interviews via Zoom with principal investigators (PIs) (*N*=30) of federally funded studies on the development and/or testing of an implementation strategy in cancer control or behavioral health. Studies funded by the NCI and NIMH that were reviewed by the Dissemination and Implementation Research in Health or the Science of Implementation in Health and Health Care study sections will be prioritized.

#### Data collection

We will review published and unpublished study protocols from PIs before interviews. Experienced qualitative interviewers (BJP and RLH) will conduct 1-h, Zoom-based interviews using a semi-structured interview guide (see Supplemental file 1). The interviews will include four major sections that provide the opportunity to (1) become better oriented to each PI’s study and the implementation strategies being tested, (2) understand the discrete components of the implementation strategies, (3) explore if and how PIs are conceptualizing and studying mechanisms in their studies, and (4) discuss their study strengths and opportunities to improve research to accelerate our understanding of how and why implementation strategies work. Before each interview, interviewers will review the PIs study protocol documents and complete a structured abstraction sheet that will be used as background information for the interview questions. Interviews will be recorded and transcribed.

#### Data analysis

After each interview, interviewers will draft field notes to record key takeaways and similarities and differences with previously conducted interviews. Transcribed interviews will be imported into NVivo [15] and analyzed using qualitative content analysis, which allows for deductive and inductive coding [16]. Deductive coding will be based upon key concepts from the interview guide (e.g., discrete strategies, mechanisms, barriers, implementation outcomes), while inductive coding will allow for additional concepts and themes to be identified. Two analysts will independently code transcripts to increase reliability and reduce bias, with a goal of at least 80% agreement. Regular meetings will be used to discuss and resolve coding discrepancies as a team throughout the coding process. Descriptive and interpretive summaries with direct quotes will be developed to support descriptions and analytic assertions. In addition to the analytic memos and co-coding of interview transcripts, we will engage in peer debriefing among the interviewers after each interview and throughout the analytic process. As we synthesize the qualitative interview findings, we will continuously update the study protocols and interviews to enhance the rigor and impact of our qualitative findings [17]. We expect this study will yield a detailed qualitative account of how NIH PIs understand implementation strategys functioning.

### Aim 1b: Develop causal pathway diagrams for 30 commonly used implementation strategies

#### Overview

To effectively enact change, implementation researchers and practitioners must have a clearer understanding of the factors required for mechanism activation as well factors that might influence strategy strength. The investigative team, which includes experts in implementation science and agile science, will develop causal pathway diagrams for 30 commonly used implementation strategies identified.

Causal pathway diagrams are an efficient way to represent evidence and hypotheses about a strategy’s operation, including the mechanisms it is intended to activate, the barrier it is intended to impact, downstream implementation outcomes that should result, and the factors that are necessary for or that moderate this causal process. Such diagrams offer initial accounts of the strategy functioning that can be further developed into robust theories of strategy operation [10]. Advances in path models and latent variable modeling allow for empirical testing of proposed causal pathways.

#### Research strategy

**Fig. 1:**
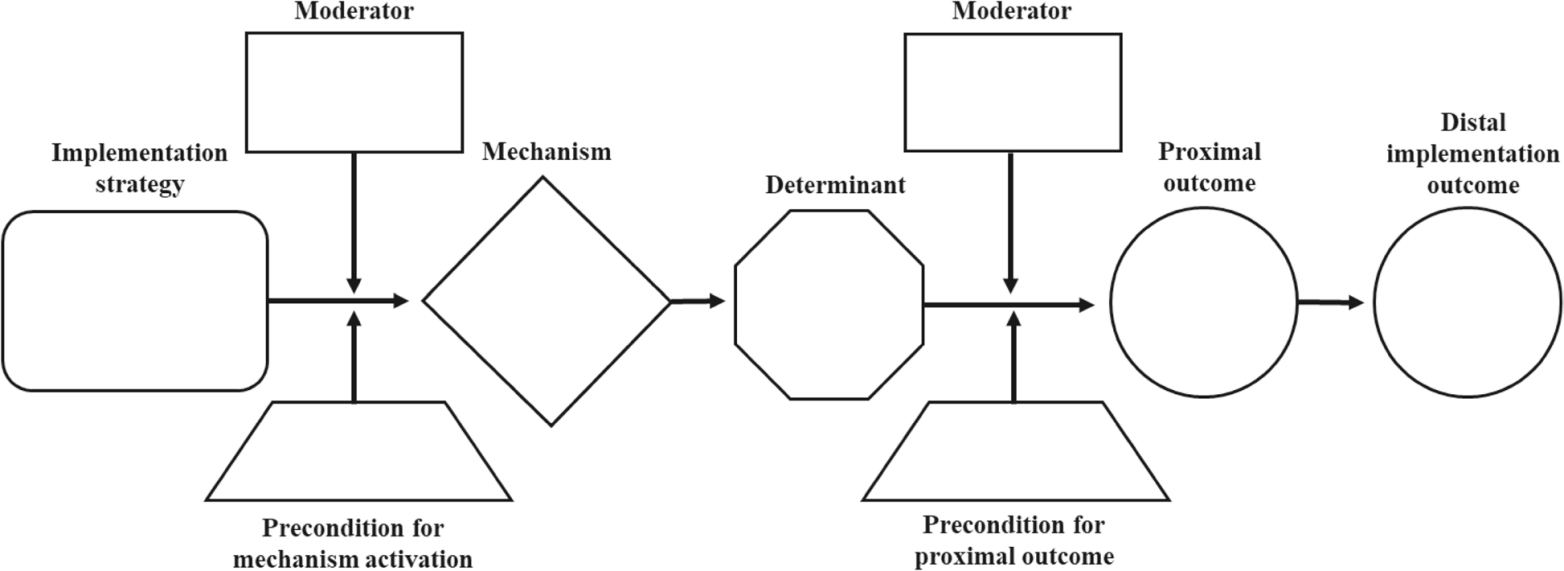
Causal pathway diagram template. Note: The number and placement of moderators and preconditions will depend on the specific causal pathway diagram being created. The placement above is merely an illustration

**Fig. 2:**
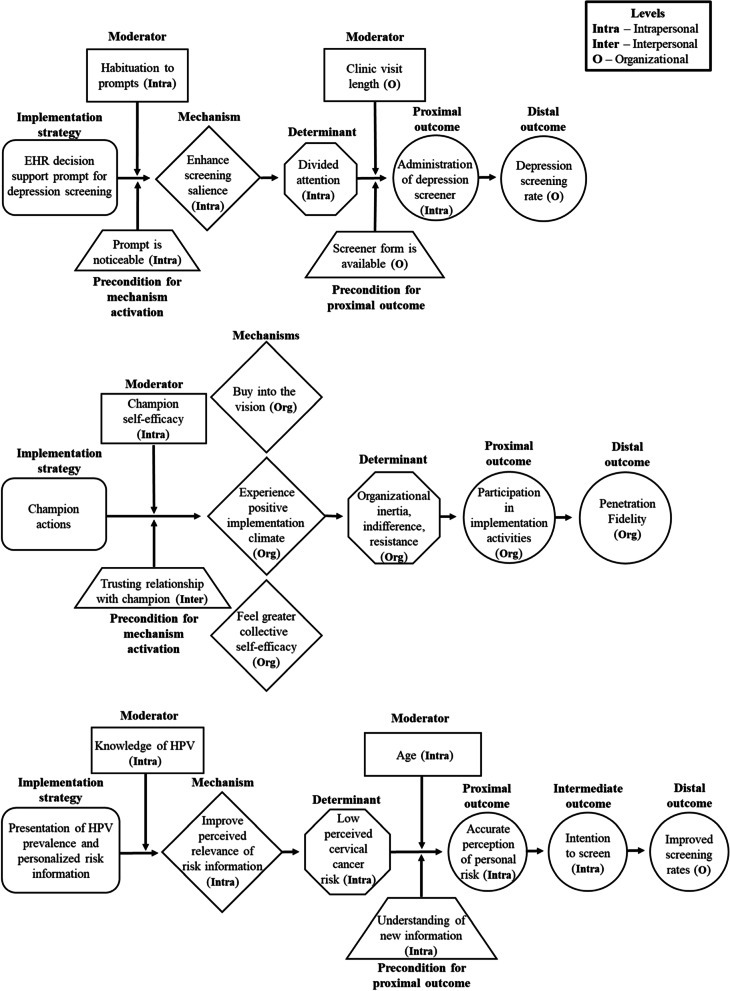
Causal pathway diagram examples. Note. *EHR* electronic health record, *HPV* human papillomavirus

We will develop sets of causal pathway diagrams for 30 strategies across five levels (patient, provider, innovation, organization, system) selected according to several criteria—these include those most commonly used by PIs interviewed in Aim 1a and level of evidence documented in systematic reviews (e.g., Effective Practice and Organization of Care) [18]. Diagrams will be developed iteratively by investigative team members using the online diagramming tool Miro [19] to allow asynchronous collaborative work on shared diagrams. Each diagram will contain these elements: (1) operationalization of an implementation strategy; (2) target barrier(s); (3) mechanism by which the strategy is hypothesized to affect the barrier; (4) observable proximal outcomes for testing mechanism activation and barrier change, which are precursors to implementation outcomes; (5) preconditions for the mechanism to be activated and to affect outcomes (6) moderators that could facilitate or impede strategy effectiveness, and (7) distal implementation outcomes that should be altered by target barrier changes (Fig. 1 includes a CPD template; Fig. 2 depicts three completed example CPDs).

Causal pathway diagram development for each strategy will (1) draw on existing theories and empirical studies of the strategy; (2) begin with implementation strategy specification; (3) articulate putative mechanisms that reflect the processes through which the strategy operates; (4) center the specific barrier(s) that a strategy can plausibly address given its mechanism of action; (5) identify the strategy’s preconditions, or factors that must be in place, for a part of the causal process to occur; (6) identify key moderators at different levels that might amplify or diminish strategy effects and depict where in the causal chain effect modification is likely to be most relevant; and (7) operationalize observable, proximal outcomes that precede implementation outcomes and provide evidence that the mechanism is being activated or that the barrier is impacted by the strategy’s administration.

For both preconditions and moderators, we will start with a list of any effect modifiers identified in existing theories and empirical literature on the strategy and theorize additional ones by applying structured prompts to those theories and studies. If a strategy is linked to multiple mechanisms, we will graph how those mechanisms and their effect modifiers might interact in the strategy’s operation by adding multiple paths in a strategy’s diagram. This sub-aim will yield causal pathway diagrams depicting strategy-mechanism-barrier-outcome linkages, preconditions, and moderators for 30 common implementation strategies.

### Aim 1c: Affirm plausibility and strength of 30 strategy-mechanism linkages

#### Overview

Aim 1c will affirm the plausibility and strength of strategy-mechanism linkages, and completeness of the CPDs, by engaging implementation scientists and other stakeholders in an expert panel process for the 30 commonly used strategies from 1b. This step ensures that the most commonly used strategies (and their associated mechanisms) are carefully vetted by subject matter experts, serving as a validation check for Aim 1b.

#### Design

Using a web-based meeting platform, we will engage nine stakeholders per strategy-mechanism linkage to rate them for plausibility and strength. After this rating, we will invite stakeholders to articulate up to three operationalizations of the strategy—specific ways that the strategy could be implemented in a particular setting. This step provides further validation of generated strategy-mechanisms linkages (i.e., checking if different operationalizations of the same strategy are feasibly activating the same mechanisms) and articulates more comprehensive sets of moderators and preconditions.

#### Participants

Each implementation strategy will be vetted by at least nine subject matter experts; the same subject matter experts could be asked to validate one or more implementation strategy-mechanism linkages and associated CPDs. We will attempt to include diverse stakeholders with respect to role (e.g., researcher, practitioner, purveyor, patient), geography, gender, and race/ethnicity. We will recruit stakeholders using multiple professional connections, such as through the Implementation Science Centers in Cancer Consortium, the Cancer Prevention and Care Research Network [20], the Mentored Training for Dissemination and Implementation Research in Cancer alumni network, the Implementation Research Hub (Ireland), the National Centre of Implementation Science (Australia), etc.

#### Strategy-mechanism-barrier ratings

Participants will have, in advance, key terms and definitions, and a standard rubric for rating strategy-mechanism plausibility and strength. Participants will first be presented with each strategy (its definition and any specification work from Aim 1a) and its putative mechanisms of action from Aim 1b to rate for plausibility and strength. Participants will rate linkages using 10-point ordinal scales from “low” to “high.” Participants will then be invited to qualitatively describe up to three different ways in which the strategy could be operationalized, inspired by prompts (e.g., delivery mode, actor, action), and then rate the causal pathway diagram for completeness using a 10-point ordinal scale. Participants will have the option to propose new moderators, preconditions, etc. and provide feedback on all variable content.

#### Expected outcomes

Practice stakeholders could use these causal pathway diagrams to plan, guide, monitor, and evaluate their EBP implementation efforts. Implementation researchers could use them to conduct mechanisms-focused implementation research by testing (i.e., validating) and refining the hypothesized linkages. Moreover, our method for developing causal pathway diagrams will be packaged into a toolkit using principles of Agile Science and housed on our website so other researchers can apply these methods to other implementation strategies.

### Aim 2: Develop reliable, valid, pragmatic measures of six implementation mechanisms

#### Overview

We will develop reliable, valid, pragmatic measures for six mechanisms identified in Aim 1. These measures will be administered across two data collection waves, an approach that was used in a previously established rapid-cycle process used by our team and recommended by experts [21–24]. The measure development and testing process involve domain delineation, survey item generation, and assessment of content validity, structural validity, known-groups validity, test-retest reliability, and sensitivity to change. We will develop pragmatic measures using a process that garners feedback from a pool of diverse stakeholders (implementation scientists, implementation-experienced cancer control practitioners, oncology nurses), ensuring brevity, readability, and relevance [25].

#### Research design: measure selection

Our measures will assess implementation mechanisms that operate at intrapersonal, interpersonal, or organizational levels, as strategies at these levels are more feasible and are more commonly deployed than those at higher levels (e.g., changing system licensure standards). In prior work [26], our team identified no psychometrically validated measures of mechanisms for the following strategies commonly used in cancer control: audit and feedback, clinical reminders, championing, multidisciplinary care teams, practice facilitation, and workflow redesign [27–31]. Provisional but plausible mechanisms for these aforementioned strategies include, respectively, highlighting performance discrepancy, cueing action (intrapersonal); promoting vision buy-in, fostering teamwork (interpersonal); and engaging in quality improvement and improving workflow (organizational).

#### Domain delineation

We anticipate that all of the 30 strategies evaluated by our team of stakeholders will include one or more theoretical constructs—that is, psychosocial and/or behavioral phenomena that are not directly measurable [23]. Domain delineation is the process of defining what a construct is and is not [22, 32]. Not only are most strategy mechanisms composed of constructs, so too are many barriers (e.g., readiness for change) and some implementation outcomes (e.g., acceptability). For each mechanism’s components, we will use its respective research literature to develop conceptual definitions, working as much as possible to distinguish it from related constructs and create a nomological network specifying causal relations (i.e., a CPD). Given that many constructs have some overlap (e.g., perceived behavioral control vs. self-efficacy) [33], we acknowledge that this will be a difficult endeavor; however,

#### Item generation

We will generate a minimum of 10–12 survey items per construct, assuming at least half will be eliminated during psychometric testing [21]. Our deductive approach to item generation [21] will use a mix of existing items from the literature and novel items that align with the conceptual definitions and nomological network.

#### Psychometric study 1

Study 1 will primarily focus on content validity, or the extent to which a measure is judged to be fully reflective of a construct of interest, be it unidimensional or multidimensional [34]. We will convene panels of content experts, one for each measure in development. Each panel will consist of 9–10 implementation scientists, cancer control practitioners, and psychometricians with experience in implementation science. Participants will be recruited from organizations such as the Implementation Science Centers in Cancer Control (ISC3) and the Cancer Prevention and Control Research Network, as well as our professional networks. Using a web-based survey, content experts will assess the content validity of items measuring one or more constructs associated with the implementation strategy mechanism of interest. Specifically, they will receive an orientation to the content validity assessment task, information about the mechanism and associated constructs, and a survey where they (a) rate the relevance of each item as an indicator of the mechanism on a four-point ordinal scale ranging from “not relevant” to “very relevant;” (b) rate the clarity of the wording of each item on a four-point ordinal scale ranging from “not clear, major revision needed” to “very clear, no revision needed;” and (c) respond to open-ended questions asking for suggested revisions to item wording, additional items, and coverage of the items as a whole (i.e., if any aspects of the mechanism not covered by the items). For each item, we will compute an Item-level Content Validity Index (I-CVI) by dividing the number of experts giving a rating of 3 (“quite relevant”) or 4 (“very relevant”) by the number of experts [35]. I-CVIs will be translated into values of a modified kappa statistic to adjust for chance agreement of content experts’ ratings [36]. Items with I-CVIs of 0.78 or higher will be considered as having acceptable content validity [36]. If content experts suggest item wording revisions or additional items, a follow-up survey containing these items will be sent to the panel for content validity assessment. Based on qualitative and quantitative analysis of the responses, the panel will identify a set of candidate measures for use in Psychometric Study 2. Items will be selected with an eye toward maximum clarity, brevity, and relevance.

#### Psychometric study 2

Study 2 assesses structural validity, reliability, known-groups validity, test-retest reliability, and sensitivity to change. Structural validity refers to the extent to which relationships among items measuring a construct accord with the construct’s expected internal structure [37].. Reliability refers to the extent to which items measuring a construct exhibit internal consistency [38]. Known-groups validity refers to the extent to which a measure is sensitive to known differences between groups [39]. Test-retest reliability refers to the extent of consistency in test scores over time. Sensitivity to change refers to the ability of a measure to detect a change in state as a change in test scores [23].

In each development wave, we will use a 2×2×2 factorial between-subjects design in which we manipulate three mechanisms at 2 opposing levels (e.g., high vs. low relative priority) in vignettes depicting a multilevel intervention to support EBP use in cancer care (e.g., integrated symptom assessment and management). We have used the vignette method successfully in prior work [40]. For each development wave, we plan to recruit, based on statistical power calculations, 240 members of the Oncology Nurse Society (ONS), an organization with 35,000+ active members working in hospital-based clinics, medical oncology units, physician offices, and other settings. We previously recruited 346 mental health counselors from a similar professional organization in a single development wave using these procedures [41]. We will compare using one-sample tests the demographics and practice characteristics of the respondents to known information about ONS members to assess for response bias. For each development wave, we will recruit from a non-overlapping sample of 1500 ONS members.

Non-retired ONS members will receive an email invitation to participate in a web-based survey, with non-respondents receiving 3 weekly reminder emails. Participants will read 1 of 8 randomly assigned vignettes. Using items generated from Psychometric Study 2, participants will indicate the extent to which the mechanism is activated from the perspective of the oncology nurse in the vignette (e.g., whether they perceive EBP use to be a relatively high or low priority, as depicted in the vignette). Participants will be re-randomized to rate either the same or the opposite vignette 3 weeks later to test for test-retest reliability or sensitivity to change, respectively. For example, a participant who received a vignette in which relative priority, teamwork, and tension for change were all designed to be high would 3 weeks later rate the vignette in which the three mechanisms were all designed to be low. We will stratify the random assignment across vignettes to ensure balance for test-retest and sensitivity analyses. Each survey is anticipated to take ~10 min, and participants will receive $25 for their efforts.

To assess structural validity, assess reliability, and identify poorly performing items, we will test separate confirmatory factor analysis models for each scale. Adequate model fit will be defined as a comparative fit index and Tucker-Lewis fit index more than 0.95 [42], standard root mean square residual less than 0.05, and RMSEA less than 0.08 [42, 43]. We will also examine factor loadings for statistical significance and adequate size (i.e., b ≥ .65). With six latent constructs and five indicators per construct (df=390), using the RMSEA test of close fit [44], with a critical alpha of 0.05, estimated power with a sample size of 240 is 100%. To assess known-groups validity we will conduct a 2^3^ analysis of covariance with the Tukey test for multiple comparisons, controlling for nurse demographics (e.g., sex as a biological variable), to determine if the mechanism scale scores varied as expected by vignette. Achieved power for this test with 240 participants and a medium effect size (*f* = 0.25) is 85.7%. Significant main effects for vignette level will indicate if the measure differentiates vignette clinics that are high or low on the mechanism. We will assess test-retest reliability by calculating a two-way mixed effects ICC (>0.70 showing reliability) between the first and second survey for participants randomized to receive the same survey. We will assess sensitivity to change using linear regression models to predict the difference score (or change in measure) based on whether the vignette assignment order between the two surveys is low-low-high, low-high-low, low-high-high, high-low-low, high-high-low, high-low-high, or high-high-high; the assignment order low-low-low will serve as the reference group.

### Aim 3: Develop and disseminate a website repository of implementation mechanisms knowledge

We propose to [1] develop a public website that hosts a knowledge repository with our CPDs, methods and associated toolkits, and measures, and (2) to actively disseminate this information to diverse stakeholders, including researchers and the practice community. The goal of our website will be to enable stakeholders to access our methods, measures, and results in ways that are useful to their own projects and research questions. Powering the public-facing website will be a relational database, structured to accommodate information contained in causal pathway diagrams, that will link (i.e., relate) information about barriers, strategies, mechanisms, outcomes, moderators, and preconditions using standardized data types, which allows for curation of knowledge over time. For example, if an organizational leader is interested in addressing a barrier to EBP implementation such as *time*, typing “time” as a barrier into a website search tool will offer information about potential strategies to address this barrier, plausible mechanisms through which these strategies operate, proximal and distal outcomes of the strategies, and preconditions and moderators affecting strategy success to consider.

#### Dissemination

We will conduct a social marketing campaign to promote public use of the website. We will conduct user research across key user segments to answer the 4 “Ps” of marketing (product, price, place, and promotion) and tailor dissemination strategies to meet diverse audiences’ needs and preferences. In terms of user research, we will inqure about what diverse users need and how our research results could meet those needs (product) as well as how to design the website and database to make those research results accessible and convenient to use (place). We will obtain additional information about how to reduce effort for diverse users to find the website (price); and how best to reach diverse users to inform them about the website and promote its use (promotion). We will work with communication experts to construct messages that appeal to different user segments to send through users’ preferred channels. We will also create a professional video overview of our products geared toward the practice community.

#### Product/website design

To design the website, we will conduct two series’ of focus groups with diverse stakeholders (one series with research and one with representatives from the practice community: providers, administrators, policy makers) to identify their information access needs. Focus groups are a commonly used needs-assessment method in user-centered design (UCD) [45, 46]. Each series will consist of three virtual sessions. The first session will introduce participants to the products of Aims 1 and 2 and elicit ideas about who might be interested in using these products and why. The expected outcome will be an initial set of personas [47, 48]—characters that represent the main types of target users for the website, acknowledging that both researchers and the practice community contain heterogeneous stakeholders. The second session will concretize the target users’ information needs by creating design scenarios [49, 50] that represent different use cases for visiting the website. To this end, participants will generate situations that different target users (represented by the personas) might encounter in their work that would motivate them to go to the website and a set of questions they would want to answer by consulting its resources. For main types of questions, participants will think aloud about how the user would want to interact with the website to get relevant information and how that information should be presented. Participants will draft brief scenarios—short narratives—that describe users’ motivations and these interactions. The third session will generate ideas for *metadata* that enable users to find what they are looking for. Using the scenarios, participants will think about the ways that the user in each scenario might try to access needed information and how results should be presented to enable finding other relevant results. This exercise will generate a preliminary list of attributes to accompany each major class of website content. The process will be repeated for the second series of focus groups. We will synthesize findings from the two groups and generate a design specification.

Using a web developer who specializes in information architecture will yield an appropriate organizational structure for the website’s database schema. In addition to its practical utility, this information architecture is a scientific contribution to the field as a way to unify and structure evidence for the operation of a wide range of implementation strategies. The web developer will design front-end functionality using the personas and scenarios.

The design will be usability-tested iteratively with target users, starting with low-fidelity prototypes [22, 23] and moving to higher-fidelity prototypes as the design matures. As is typical in UCD, evaluations will focus on comprehensibility, perceived usefulness, usability, and user satisfaction. These characteristics will be assessed using qualitative interviews, task-based prototype walk-throughs [23], and, in the final evaluation, a set of standard usability scales [25–27]. The evaluation results will be used to iteratively revise the design to optimize the website’s usefulness and ease of use. After review of all user data, the website will be refined and ready for dissemination.

## Discussion

Despite advances in implementation barriers [51], models and frameworks [52, 53], outcomes [54], and in evaluating the general effectiveness of strategies [55], implementation mechanisms are underdeveloped and understudied [9]. The robust methods and measures described herein will facilitate mechanisms-focused implementation research, opening new horizons in implementation strategy development, optimization, evaluation, and deployment [56]. Strategy-mechanism linkages and full causal pathway diagrams will improve the design of implementation strategies and generate more robust evidence about which strategies work best in which contexts. Multicomponent and multilevel strategies could be optimized by focusing on those elements that best engage key mechanisms. When implementation strategies fail, scientists could investigate *why* by examining if the strategy failed to engage key mechanism(s), or if a contextual factor moderated flow of the effect from the strategy to the mechanism(s) to the outcome [57]. These advances in implementation science, in turn, could guide practitioners in selecting strategies optimized to address specific problems. Practitioners could use our reliable, valid, pragmatic measures of mechanisms to detect early if a strategy is working. Our use of UCD principles to build the website repository of implementation mechanisms knowledge combined with social marketing to actively engage intended users in our website increases our potential for impact.

## Supplementary Information

**Additional file 1.** Aim 1a Interview Guide.

## Data Availability

Data sharing is not applicable to this article as no datasets were generated or analyzed during the current study.

## Abbreviations

EBP: Evidence-based practice
EHR: Electronic health record
CPD: Casual pathway diagrams
HPV: Human papillomavirus
ICC: Intraclass correlation coefficient
I-CVI: Item-level Content Validity Index
ISC3: Implementation Science Centers in Cancer Control
NCI: National Cancer Institute
NIH: U.S. National Institutes of Health
NIMH: National Institute of Mental Health
ONS: Oncology Nurse Society
PIs: Principal investigators
RMSEA: Root mean square error of approximation
UCD: User-centered design
