# Supplementary material for: The mechanics of implementation strategies and measures: advancing the study of implementation mechanisms
Source: Implement Sci Commun. 2022 Oct 22;3:114. doi: 10.1186/s43058-022-00358-3 (PMC9588220; doi:10.1186/s43058-022-00358-3)
Supplement: Supplementary file 1 — Additional file 1. Aim 1a Interview Guide. [file 43058_2022_358_MOESM1_ESM.pdf]

Interviewer Names: ______________________________ Participant ID: _______________________
Date of Interview: ___ (DD) / ___ (MM) / ______ (YYYY) Start time: ___:___ End time: ___:___

Aim 1a Interview Guide

(Last revised: 5/24/2022)

Thank you for speaking with us today. You may remember from our initial email to you that you were contacted for this study because we are conducting interviews with individuals who are currently engaged in a trial of implementation strategies or who have recently completed one. We are interested in how people think about mechanisms.

As we move through the interview, it is important to note that there are no right or wrong answers. You are not required to answer our questions, and you may skip any questions that make you uncomfortable. If you decide that you no longer want to participate in this interview, it will not affect your status in our study.

We will record our conversation and it will later be transcribed. We plan to report themes across participants and de-identify quoted material. However, if a particularly illustrative, concrete example emerges in our interview, we may wish to include this in published material. Although we will not attribute information to an individual, there is a chance that this information could be identifying given the specifics of a particular project (e.g., the name or nature of the implementation strategy studied). If this is the case, we will engage in member-checking with you prior to publication to ensure that the information is presented in acceptable way.

The Washington University IRB has reviewed and approved this exempt study. Do you have any questions before we begin the interview?

BEGIN RECORDING

**Main Questions**	**Probes**
**Getting oriented to the implementation strategy (or strategies)**	
1. We had a chance to review your study protocol, but would you mind giving us a brief update on what stage the study is in?	
2. What are the gaps your study is trying to fill related to implementation science?	
3. Could you please briefly describe the study conditions / intervention comparison groups?	
4. Why did you choose this implementation strategy (or strategies)?	What problem were you trying to resolve? [barriers]<br><br>Were there any theories or conceptual frameworks informing this?
**Understanding the individual strategy components**	
Strategies are often multifaceted and/or multilevel. We are interested in how you identified and operationalized the discrete or component strategies within your overarching strategy.	

5. Could you tell us a bit more about these discrete components?	
6. Who chose these discrete components? How?	
7. What outcomes were you trying to improve or determinants were you trying to address by choosing these strategies?	Did you measure those things? If so, how?
8. How were the discrete components intentionally bundled?	In other words, if studying a set of strategies, how did you expect them to work together?
**Mechanisms**	
9. Broadly speaking, how do you define mechanisms?	
10. At the time you conceptualized the study, did you give some thought about the mechanism or mechanisms of the strategy (or set of strategies)?	**[If they say yes]**<br><br>Great! Can you tell us about how you conceptualized mechanisms within your study?<br><br>Did you explicitly study the mechanism(s) (e.g., qual, quant, mixed methods)?<br><br>**[If they say no]**<br><br>Great, we have time to talk about that now!<br><br>Thinking about your study now, how might you think about mechanisms?
Mechanisms can operate at different levels of influence in the social ecological model, e.g., individual, interpersonal, organizational, etc.<br><br>11. At what levels do you think the mechanism(s) associated with these strategies operate?	
12. Is there evidence, theory, practical experience, or other information that led you to believe that the strategy would operate through those mechanisms?	
13. Were you expecting certain contextual factors to influence if or how these mechanisms would be activated?	
14. (**If relevant**) Did the mechanisms work as you expected? How do you know?	If not, why? What did you learn about other potential mechanisms?

Future work	
15. What do you think you did particularly well with respect to the identification and evaluation of implementation mechanisms in your study? What might you do differently in a future study?	What would you measure?<br><br>What research designs would you use?<br><br>What stakeholders would you engage or learn from?
16. What can we do as a field to better understand how and why implementation strategies work?	

Thank you so much for your time today. As a token of our gratitude, we would like to provide you with at $50 Amazon gift card.

Washington University requires that we request your social security number for tax purposes. Are you comfortable providing that to me?

What is the best email to send your gift card?
